# Silencing of NOTCH3 Signaling in Meniscus Smooth Muscle Cells Inhibits Fibrosis and Exacerbates Degeneration in a HEYL‐Dependent Manner

**DOI:** 10.1002/advs.202207020

**Published:** 2023-04-07

**Authors:** Hao Sun, Fangzhou Liu, Zhencan Lin, Zongrui Jiang, Xingzhao Wen, Jie Xu, Zhiqi Zhang, Ruofan Ma

**Affiliations:** ^1^ Department of Orthopaedic surgery Sun Yat‐sen Memorial Hospital Sun Yat‐sen University Guangzhou Guangdong 510120 China; ^2^ Department of Joint Surgery First Affiliated Hospital of Sun Yat‐sen University Guangzhou Guangdong 510080 China

**Keywords:** meniscus degeneration, meniscus fibrosis, NOTCH3, smooth muscle cell

## Abstract

The mechanisms of meniscus fibrosis and novel ways to enhance fibrosis is unclear. This work reveals human meniscus fibrosis initiated at E24 weeks. Smooth muscle cell cluster is identified in embryonic meniscus, and the combined analysis with previous data suggests smooth muscle cell in embryonic meniscus as precursors of progenitor cells in the mature meniscus. NOTCH3 is constantly expressed in smooth muscle cells throughout embryogenesis to adulthood. Inhibition of NOTCH3 signaling in vivo inhibits meniscus fibrosis and exacerbates degeneration. Continuous histological sections show that HEYL, NOTCH3 downstream target gene, is expressed consistently with NOTCH3. HEYL knockdown in meniscus cells attenuated the COL1A1 upregulation by CTGF and TGF‐*β* stimulation. Thus, this study discovers the existence of smooth muscle cells and fibers in the meniscus. Inhibition of NOTCH3 signaling in meniscus smooth muscle cells in a HEYL‐dependent manner prevented meniscus fibrosis and exacerbated degeneration. Therefore, NOTCH3/HEYL signaling might be a potential therapeutic target for meniscus fibrosis.

## Graphical abstract

Smooth muscle cells are first identified in the meniscus. Characteristics of smooth muscle fiber in meniscus is elucidated and smooth muscle cells is discovered as precursors of fibrochondrocyte progenitors. NOTCH3 inhibition in meniscus smooth muscle cells prevents meniscus fibrosis and exacerbated degeneration in a HEYL‐dependent manner.

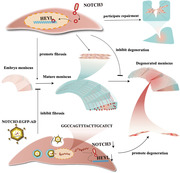

## 1 Introduction

During human limb development, the meniscus originates from the interzone between the tibia and the femur, and the typical meniscus structure is formed from weeks 7 to 8 of embryonic development.^[^ ^1^ ^]^ In the beginning of this process, the meniscus is fully vascularized, and as it develops, the meniscus loses vascularization to contain blood vessels only in its peripheral region.^[^ ^2^ ^]^ Along with decreased blood supply, the fibrotic area of the meniscus gradually increases.^[^ ^3^ ^]^ The mature meniscus is a completely fibrotic tissue, playing an important role in joint stability, shock absorption, and distribution of contact forces.^[^ ^4^ ^]^ However, the regulatory mechanisms that drive human meniscus fibrosis remain unclear.

There are several cells in the mature meniscus with different morphologies and phenotypes; however, the cell types present in this structure are controversial. A recent study divided meniscus cells according to their distribution: fibroblast like cells in the outer zone, chondrocytes like cells in the inner zone, and possibly progenitor cells in the superficial zone.^[^ ^5^ ^]^ Our recent study showed that the meniscus has a far more complex cellular structure, and we characterized the cellular phenotype and functional characteristics of the meniscus progenitor cells.^[^ ^6^ ^]^ Nevertheless, the precursors of mature meniscus progenitors and the effect on meniscus fibrosis remain unknown.

The meniscus is a highly hydrated structure, with 72% water and 28% dry weight. Collagen accounts for the majority of the dry weight, and human meniscus degeneration involves dry weight decrease and wet weight increase.^[^ ^7^ ^]^ In the meniscus, collagen fibers are oriented circumferentially in the inner zone and radially in the superficial zone.^[^ ^8^ ^]^ Using single‐cell RNA sequencing, we identified more cell types in the meniscus than previously reported^[^ ^6^ ^]^; however, fiber structure and their corresponding function remained to be elucidated.

In this study, we investigated the regulatory mechanisms involved in the initiation of meniscus fibrosis, exploring potential therapeutic targets for fibrosis promotion and degeneration prevention. We delineated an atlas of human meniscus cells at fibrosis initiation using single‐cell sequencing and found that smooth muscle cells in embryonic meniscus are the precursor cells of fibrochondrocyte progenitors in the mature meniscus, determinant for fibrosis. In vivo NOTCH3 inhibition impaired meniscus fibrosis by smooth muscle cells repression.

## 2 Results

### 2.1 The Fibrosis of Human Meniscus Emerged at Embryonic 24 Weeks

**Figure 1:**
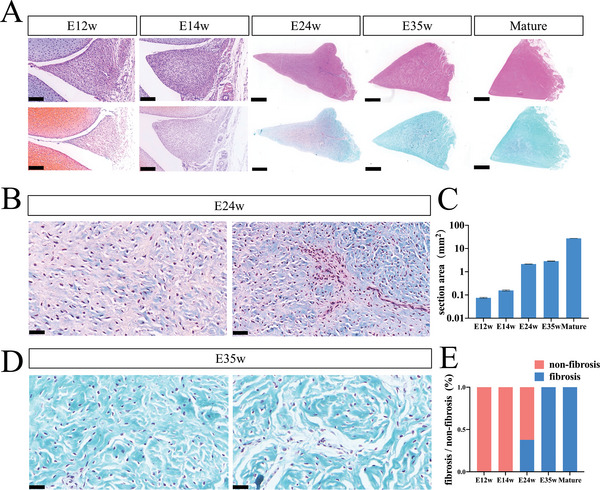
General morphology of embryo meniscus at different stages of development. A) HE (hematoxylin‐eosin) and SafraninO (S/O)‐staining of embryo meniscus cross‐section in E12w (weeks), E14w, E24w, E35w, and healthy meniscus. Scale bar: 50 µm for E12 and E14 weeks; 200 µm for E24 and E35 weeks; 1000 µm for mature meniscus. *n* = 3. B) S/O‐staining of different meniscus areas showing fibrosis progression at E24w. Scale bar: 20 µm. *n* = 4. C) Section area count of different stages demonstrates increasing section area. D) S/O‐staining of different areas showing fibrosis progression in E35w. Scale bar: 20 µm. *n* = 4. (E) Fibrosis area and non‐fibrosis area ratio of S/O staining of different stages.

The mice meniscus formed distinct structure at embryonic 16 days (E16) while fibrotic area appeared on the seventh day after birth.^[^ ^6^, ^9^ ^]^ To investigate the fibrosis process of human meniscus, we performed the histology of human embryonic and mature meniscus. HE staining showed the formation of distinct meniscus structures at embryonic week 12 (E12 week) (**Figure** **1**A). Safranin O/Fast green staining showed fibrosis initiation at E24 weeks concomitant with vessel formation (Figure 1B). With increasing cross‐sectional area (Figure 1C), the fibrotic areas gradually increased until totally fibrotic at E35 weeks, a feature maintained into adulthood (Figure 1D,E).

### 2.2 scRNA‐seq of Human E24 and E35 Weeks Meniscus Identified Three Distinct Cell Populations

**Figure 2:**
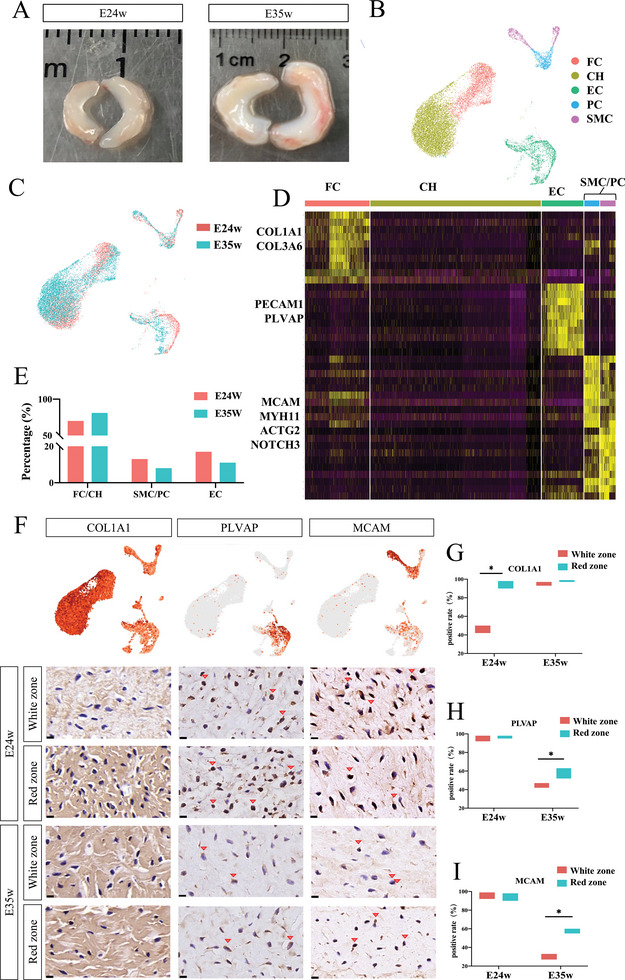
Heterogenicity of meniscus cells and spatiotemporal characterization of specific cell clusters. A) Morphology of embryo meniscus at E24w and E35w. B) Uniform manifold approximation and projection (UMAP) results for major cell clusters in the E24w and E35w meniscus. C) Sample distribution of E24w and E35w in UMAP. D) Heatmap of significant marker genes for major cell clusters: endothelial cell (EC) (PLVAP+, PECAM1+, and NOTCH1), smooth muscle cell/pericyte (SMC/PC) (MCAM+, NOTCH3+, and MYH11+), fibrochondrocyte (FC) and chondrocyte (CH) (COL1A1+ and COL6A1+). E) Major cell clusters in the E24w and E35w meniscus show EC and SMC/PC decrease and FC/CH increase. F) Immunohistochemistry staining for COL1A1, PLVAP, and MCAM in white zone and red zone of E24 and E35 weeks meniscus and their distribution in UMAP. Scale bar; 10 µm. *n* = 5 G) Quantification of COL1A1 positive area at E24w and E35w. At E24w, COLA1 positive rate was 45% in white zone and almost 100% in red zone, whereas in E35w both white and red zone were almost 100% COL1A1 positive. *n* = 5, *p < 0.05. H) Quantification of PLVAP positive cells at E24w and E35w. At E24w, PLVAP positive rate was 87% in white and red zone, whereas in E35w it decreased to 42% in white zone and 54% in red zone. *n* = 5, *p < 0.05. I) Quantification of MCAM positive cells at E24w and E35w. At E24w, positive rate was nearly 100% in both white and red zone. At E35w, the positive rate in white zone declined to 30% and nearly 60% in red zone. *n* = 5, *p < 0.05.

Single‐cell RNA sequencing at E24 and E35 weeks allowed us to explore the cellular mechanisms of fibrosis (**Figure** **2**A). We obtained satisfactory cell quality for scRNA‐seq (Figure S1, Supporting Information). Unbiased clustering of meniscus cells resulted in three general clusters (Figure 2B,C): fibrochondrocyte/chondrocyte (FC/CH, expressing COL1A1 and COL3A1), endothelial cell (EC, expressing PECAM1 and PLAVP), and smooth muscle cell/pericyte (SMC/PC, expressing ACTG2 and NOTCH3) (Figure 2D). At E35 weeks, the number of FCs/CHs was elevated (69.7% to 81.1%) while the ECs (17.2% to 11.1%) and SMCs/PCs (13.1% to 7.8%) was decreased (figure 2E), compared with that at E24 weeks. Consistent with these findings, the immunohistochemistry (IHC) staining of E35 weeks tissue showed upregulated COL1A1 levels and downregulated PLVAP and CD146 (encoded by MCAM) levels (Figure 2F–I).

The meniscus can be divided into: outer red zone (vascular region), inner white zone (avascular region), and the red‐white zone between both.^[^ ^4^ ^]^ Using IHC, we analyzed the distribution of each cellular cluster within the meniscus. COL1A expression was higher in the red zone than in white zone at E24 weeks (Figure 2G), while no differences were observed at E35 weeks, indicating that the red zone may be the first region to undergo fibrosis. In contrast, PLVAP and MCAM were higher in red zone than in white zone at E35 weeks, showing no difference at E24 weeks (Figure 2H,I), illustrating the differentiated blood supply at this stage.

### 2.3 Identification of Smooth Muscle Structures in the meniscus

**Figure 3:**
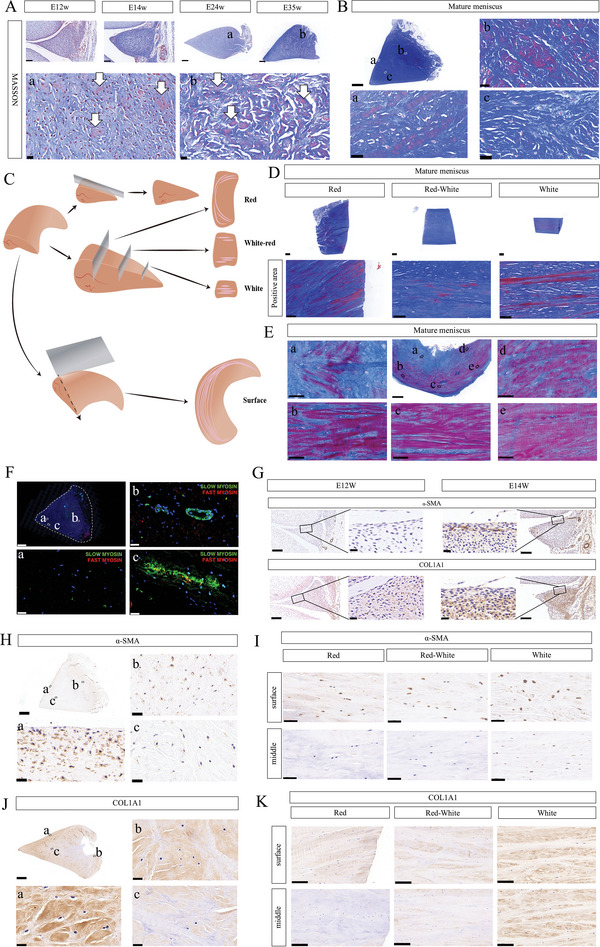
Identification of smooth muscle structures in meniscus. A) Masson staining of E12w, E14w, E24w, and E35w shows smooth muscle fibers. Scale bar: 50 µm for E12w and E14w; 200 µm for E24w and E35w; 10 µm for magnified image. *n* = 3. B) Masson staining of mature meniscus shows smooth muscle fibers. Scale bar: 1000 µm for mature meniscus; 10 µm for magnified image. *n* = 4. C) Diagrammatic sketch of the sliced meniscus in sagittal or coronal sections. Different slices from coronal section named according to location as Red, Red‐White, and White. D) Masson staining positive areas in coronal sections of the meniscus. Scale bar: 1000 µm for overall view; 50 µm for positive area of Masson staining. *n* = 4. E) Masson staining in different areas in horizontal section of meniscus. Scale bar: 4000 µm for overall view; 100 µm for magnified picture. *n* = 3. F) Immunofluorescence (IF) of fast and slow myosin confirmed the existence of smooth muscle fiber in the meniscus. Scale bar: 1000 µm for overview; 20 µm for magnified picture. *n* = 3. G) Immunohistochemistry (IHC) of *α*‐SMA and COL1A1 of E12w and E14w meniscus reveals co‐localization of *α*‐SMA, COL1A1. Scale bar: 50 µm; 10 µm for magnified picture, *n* = 3. H) IHC of *α*‐SMA in sagittal meniscus sections. Scale bar: 1000 µm for overall view; 20 µm for magnificent view. *n* = 3. (I) IHC of *α*‐SMA in different parts of a coronal section of the meniscus. Scale bar: 50 µm. *n* = 6. J) IHC of COL1A1 staining in a sagittal section of the meniscus. Scale bar: 1000 µm for overall view; 50 µm magnified image. *n* = 3. K) IHC of COL1A1 staining in different parts of a coronal section of the meniscus. Scale bar: 50 µm. *n* = 6.

Our scRNA‐seq results showed the presence of numerous smooth muscle cells in the meniscus, but these results were not reported in previous studies. Therefore, we further identified the presence of smooth muscle tissue in meniscus, as well as its distribution characteristics. Masson trichrome staining allows differentiation between smooth muscle tissue and collagenous fiber tissue, staining the smooth muscle cells red and the collagenous fiber tissue blue.^[^ ^10^ ^]^ Masson staining revealed that smooth muscle tissue initially emerged at E24 weeks (**Figure** **3**A), and this structure was even more distinct in the mature meniscus (Figure 3B).

Next, we used different histological slicing methods to investigate distribution of smooth muscle fibers in the mature meniscus (Figure 3C). Typical smooth muscle fibers were observed in coronal slices (Figure 3C) but not in sagittal slices (Figure 3B). In coronal slices of the red, red‐white, and white zones, the smooth muscle fibers were scattered around the basal and outer area of the meniscus and were less distributed in the inner area (Figure 3D). Slices along surface of meniscus was also managed to better interpreted smooth muscle fiber (Figure 3E). Myosin, an important structure for smooth muscle contraction, can be divided into fast and slow myosin.^[^ ^11^ ^]^ Co‐immunofluorescence of both myosin forms showed distribution of slow myosin on the meniscus surface and perivessels, while no fast myosin was detected (Figure 3F).

*α*‐SMA (alpha‐smooth muscle actin) is the classical marker for smooth muscle cells,^[^ ^12^ ^]^ therefore we performed IHC staining of *α*‐SMA in our meniscus samples. *α*‐SMA positive cells were first found at E14 weeks, mainly distributed at the base and surface layer of the meniscus (Figure 3G). COL1A1 positive cells were also first detected at this time (Figure 3G). At E24 and E35 weeks, *α*‐SMA positive cells were widely distributed throughout the meniscus, and the fibrosis process was launched during this stage (Figure S2, Supporting Information). In mature meniscus, *α*‐SMA positive cells were mainly distributed at the base and surface layer, consistent with COL1A1 and smooth muscle fibers distribution (Figure 3H–K).

Altogether, these results demonstrated the existence of smooth muscle tissue in human meniscus and also revealed the time and spatial consistency between smooth muscle cells/fibers and COL1A1 expression, suggesting that smooth muscle cells/fibers play an important role meniscus fibrosis process.

### 2.4 Embryonic Meniscus Smooth Muscle Cells are the Precursors of Fibrochondrocyte Progenitors of the Mature Meniscus

**Figure 4:**
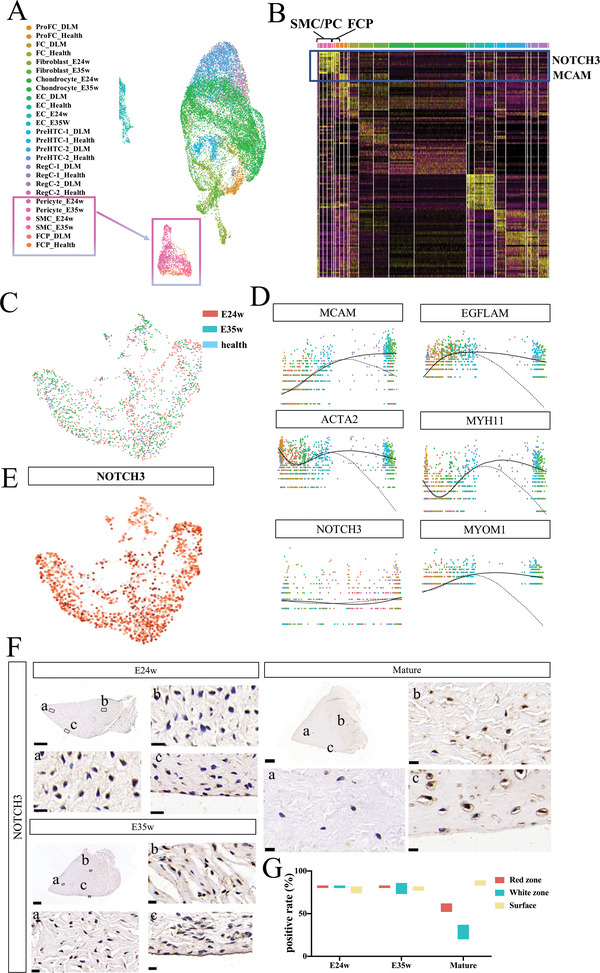
Precursors of mature meniscus progenitor cells in embryo are smooth muscle cells. A) Uniform manifold approximation and projection (UMAP) results of co‐analysis between meniscus cells in E24w, E35w, healthy adult meniscus, and discoid meniscus. B) Significant marker genes of cell clusters in the co‐analysis of meniscus cells at E24w, E35w, healthy adult meniscus, and discoid meniscus. Smooth muscle cell/pericyte (SMC/PC) and fibrochondrocyte progenitors (FCP) present constant expression of NOTCH3, MCAM, ACTG2, and COL4A1. C) Sample distribution in UMAP results of SMC/PC from embryo and FCP from health meniscus. D) MCAM, EGFLAM, ACTA2, MYH11, NOTCH3, and MYOM1 distribution in pseudo time kinetics analysis reveals a constant staining of NOTCH3 through this trajectory. The active line indicates cell fate for SMC/PC in embryo, and the dotted line indicates cell fate for FCP in adult meniscus. E) NOTCH3 distribution in UMAP result demonstrates widely expression in these sub‐clusters. F,G) Immunohistochemistry (IHC) of NOTCH3 distribution (F) in E14w, E24w, E35w, and mature meniscus and quantification of NOTCH3 positive rate (G) in different areas for each group. Scale bar: 1000 µm for overview, 10 µm for magnified view. *n* = 4.

Our previous study identified seven different cell clusters of healthy meniscus cells, including fibrochondrocyte progenitors (FCP), endothelial cells (EC), and five parenchymal cell clusters.^[^ ^6^ ^]^ To investigate the correlation between embryonic smooth muscle cells and mature meniscus cells, we combined our previous healthy adult meniscus cell^[^ ^6^ ^]^ and adult discoid meniscus cells^[^ ^13^ ^]^ scRNA‐seq data with the embryonic meniscus scRNA‐seq data. After merging, the uniform manifold approximation and projection (UMAP) plot^[^ ^14^ ^]^ showed that all meniscus cells could be defined as three general clusters: fibrochondrocyte/chondrocyte (FC/CH), endothelial cell (EC), and smooth muscle cell/pericyte (SMC/PC) (**Figure** **4**A, Figure S3A,B, Supporting Information). FCP was highly homologous to the embryonic smooth muscle cells (Figure 4A, Figure S4A–C, Supporting Information), with consistent expression of marker genes^[^ ^15^ ^]^ such as MCAM, NOTCH3, and ATCG2 (Figure 4B), indicating that the smooth muscle cells at the embryonic stage were the precursors of the FCP in the mature stage.

Next, we extracted data on SMC/PC from individual single‐cell sequencing samples (E24 weeks, E35 weeks, and healthy meniscus) and performed additional analyses. SMCs from each sample were evenly distributed in different sub‐clusters (Figure 4C, Figure S3C,D, Supporting Information), suggesting that smooth muscle cells maintain a relatively stable phenotype during meniscus development.

We used the Monocle method to reconstruct the pseudospace trajectory of smooth muscle cells, containing one root and two termini corresponding to origin and two distinct cell fates, respectively (Figure S3E–G, Supporting Information). The expression of most smooth muscle cell marker genes varied greatly from root to the two termini, except for NOTCH3, with a constant expression throughout the entire trajectory (Figure 4D,E). IHC staining showed that NOTCH3 levels were mainly distributed on the surface of the meniscus, similarly to *α*‐SMA and COL1A1 (Figures 4F,G, Figure S4, Supporting Information). Therefore, NOTCH3 was used as the gene marker for smooth muscle cells in subsequent experiments.

### 2.5 NOTCH3 Knockdown In Vivo Impaired Meniscus Fibrosis

**Figure 5:**
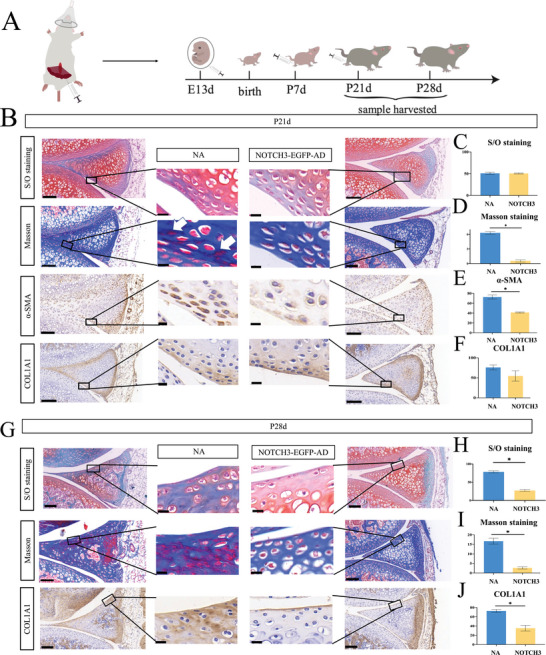
NOTCH3‐knockdown in mice impairs meniscus fibrosis progression. A) Diagrammatic sketch of the study design. B) SatraninO (S/O)‐staining, Masson staining, and immunohistochemistry (IHC) of *α*‐SMA and COL1A1 in meniscus of 21‐day‐old mice after NOTCH3‐knockdown. Scale bar: 50 µm; 5 µm for magnified picture. *n* = 6. C) S/O staining quantification indicates no significant differences observed in P21d, *n* = 3. D) Masson staining positive rate quantification shows significant differences. Magnified view indicates vanishing of smooth muscle fiber after NOTCH3 knockdown. *n* = 3, *p < 0.05. E) IHC of *α*‐SMA shows significant decrease in positive rate, decreased to 40% after NOTCH3 knockdown, *n* = 3, *p < 0.05. F) COL1A1 expression quantification presented no significant differences in P21d, *n* = 3. G) S/O‐staining, Masson staining, and IHC of COL1A1 in meniscus of 28‐day‐old mice after NOTCH3‐knockdown. Scale bar: 100 µm; 10 µm for magnified picture. *n* = 4. H–J) S/O staining (H), Masson staining (I), and COL1A1 IHQ (J) all show the same tendency, with a decline in the positive rate after NOTCH3 knockdown, *n* = 3, *p < 0.05.

To investigate the effect of smooth muscle cells on meniscus fibrosis progression, we constructed a mouse model of NOTCH3 knockdown in meniscus. NOTCH3‐EGFP‐AD adenovirus, to achieve concomitant NOTCH3 knockdown and EGFP expression, were injected in amniotic the cavity pregnant mice at E13, and in knee joint at P7 and P21 (**Figure** **5**A) (since knockdown could only be achieved for 2 weeks). Mice were sacrificed at P21 and P28. Immunofluorescence staining showed that NOTCH3‐EGFP‐AD successfully invaded the meniscus and significantly decreased NOTCH3 expression. Mice body length and weight were not affected by this approach (Figure S5, Supporting Information).

Although Safranin O/Fast green and COL1A1 staining showed identical fibrosis, Masson staining suggested that smooth muscle fibers were significantly reduced with NOTCH3 knockdown. Furthermore, *α*‐SMA levels were also significantly downregulated with NOTCH3 knockdown in mice meniscus, compared with those in P21 control group (Figure 5B–F). At P28, smooth muscle fibers were still reduced, Safranin O/Fast green staining showed a delayed fibrosis process, and the COL1A1 levels were decreased compared with those in the control group (Figure 5G–J). Collectively, *α*‐SMA expression and smooth muscle fibers were impacted in an earlier stage than COL1A1 levels, suggesting that NOTCH3 inhibition may repress meniscus fibrosis through regulation of smooth muscle cells.

### 2.6 NOTCH3 Inhibition In Vivo Accelerated Meniscus Degeneration

**Figure 6:**
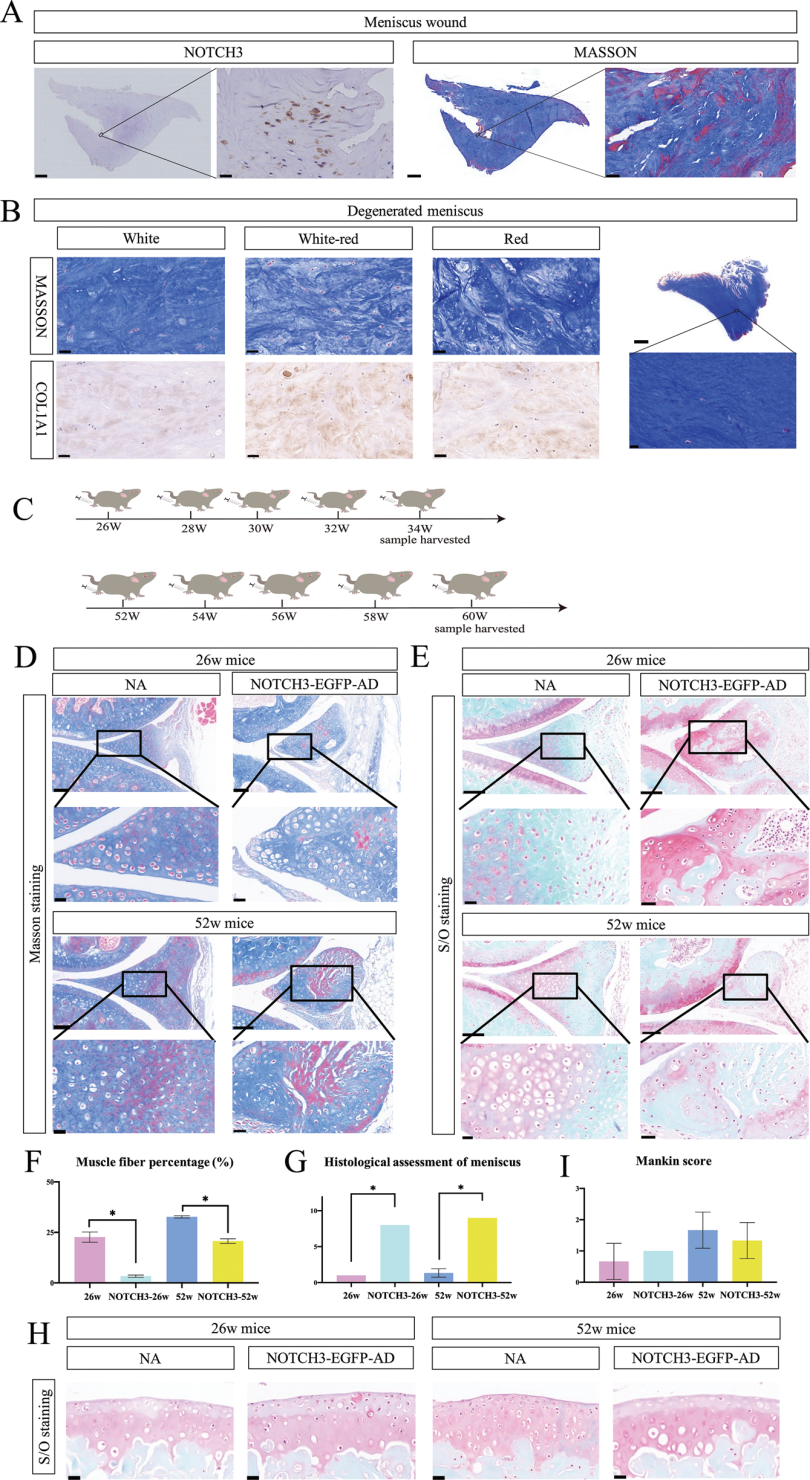
NOTCH3‐knockdown in mice accelerates meniscus degeneration. A) Immunohistochemistry (IHC) of NOTCH3 and Masson staining of a wound in human meniscus indicated that smooth muscle fiber composed of smooth muscle cell (SMC) marked by NOTCH3 take part in meniscus repair. Scale bar: 1000 µm; 20 µm for magnified picture. *n* = 3. B) IHC of COL1A1 and Masson staining of degenerated human meniscus showing no muscle fibers. Scale bar: 50 µm for broad‐section; 1000 µm for overview of degenerated meniscus, 20 µm for magnified picture. *n* = 6. C) Diagrammatic sketch of study design. D) SatraninO (S/O) and Masson staining of meniscus from 26 weeks‐old mice with 8 weeks of intraarticular injection of NOTCH3‐EGFP‐AD versus that in the control group. After injection, meniscus presents progressive degeneration with disorganized collagen fiber and decreased smooth muscle fibers. Tibia and femoral side show no significant differences. Scale bar: 100 µm; 20 µm for magnified picture. *n* = 5. E) S/O and Masson staining of meniscus from 52 weeks mice with 8 weeks of intraarticular injection of NOTCH3‐EGFP‐AD versus that of the control group. Scale bar: 100 µm; 20 µm for magnified picture, *n* = 6. F) Masson staining positive rate for 26w and 52w declines after NOTCH3 knockdown, *n* = 6, *p < 0.05. G) Histology assessment (histological score) of meniscus shows a severe degeneration grade upon NOTCH3 knockdown, *n* = 6, *p < 0.05. H) S/O staining of articular cartilage in different samples. Scale bar: 20 µm, *n* = 6, *p < 0.05. I) Mankin score for different stages shows no significant differences between groups, *n* = 6, *p < 0.05.

Our previous study demonstrated that the FCP originating from smooth muscle cells promoted mouse meniscus injury repair.^[^ ^6^ ^]^ In our present study, we obtained human injured meniscus from a total knee arthroplasty surgery. In this tissue, *α*‐SMA levels and smooth muscle fibers were increased in repairing areas, indicating that smooth muscle cells also participate in meniscus injury repair in humans (**Figure** **6**A). Smooth muscle fibers and COL1A1 levels were remarkable reduced in the human degenerated meniscus (Figure 6B).

In a subsequent experiment, we performed knee joint NOTCH3‐EGFP‐AD injections in 26 and 52 weeks old mice every 2 weeks. Mice were sacrificed 8 weeks after the first injection to evaluate NOTCH3 inhibition effect on meniscus degeneration (Figure 6C). NOTCH3 inhibition led to disorganization of meniscus fibers and cell hypertrophy at 26 and 52 weeks (Figure 6D,E). Histological assessment^[^ ^16^ ^]^ showed more severe degeneration grade when compared to that in the control (Figure 6F,G). IHC of MYOCD was performed to demonstrated decreasing smooth muscle characteristics in NOTCH3 inhibition mice meniscus (Figure S6A, Supporting Information). Paw withdrawal threshold also indicated aggravated pain after NOTCK3 inhibition (Figure S6B, Supporting Information). However, cartilage degeneration was not accelerated during this period (Figure 6H,I).

### 2.7 NOTCH3 Downstream Target HEYL is Required for Meniscus Fibrosis

**Figure 7:**
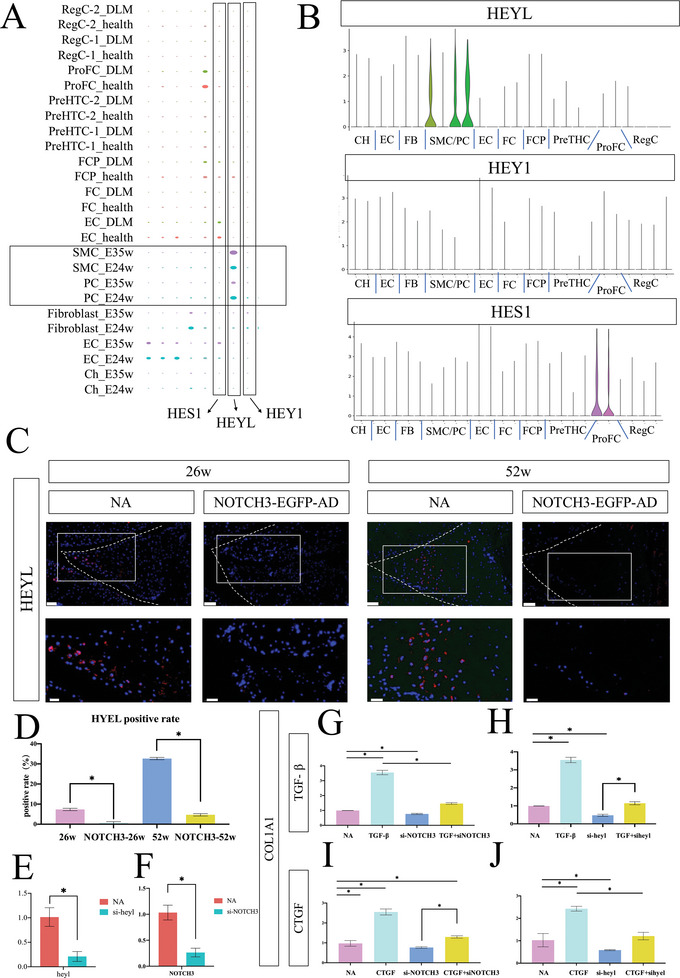
NOTCH3 downstream target HEYL is required for meniscus fibrosis. A) Gene bubble plot reveals HEYL as the most relevant downstream gene associated with the embryo meniscus. During development, HEYL expression is declined, which indicates a correlation between HEYL and COL1A1 expression. B) Vin‐plot of all clusters and HEYL, HEY1, and HES1 expression. HEYL is specifically expressed in smooth muscle cell/pericyte (SMC/PC) from embryo. C) Immunofluorescence (IF) of meniscus indicates lower expression of HEYL after NOTCH3 knockdown. Dotted line indicates the board of meniscus. Scale bar: 50 µm; 20 µm for magnified picture. *n* = 3. D) HEYL positive rate declines after NOTCH3 knockdown, *n* = 3, *p < 0.05. E,F) mRNA expression of NOTCH3 (E) and HEYL (F) after knockdown of NOTCH3 (E) or HEYL (F), respectively, *n* = 3, *p < 0.05. G,H) Human healthy meniscus cells subjected to: TGF‐*β* (40 ng mL^−1^) for 48 h, NOTCH3 knockdown, and combined NOTCH3 knockdown with TGF‐*β* (40 ng mL^−1^). Negative siRNA was used in the control group, *n* = 3, *p < 0.05. I,J) Human healthy meniscus cells subjected to: CTGF (40 ng mL^−1^) for 48 h, NOTCH3 knockdown, and combined NOTCH3 knockdown with CTGF (40 ng mL^−1^). Negative siRNA was used in the control group, *n* = 3, *p < 0.05. G,I) NOTCH3 knockdown attenuates TGF‐*β*‐induced (G) and CTGF‐induced COL1A1 upregulation, *n* = 3, *p < 0.05. H,J) HEYL knockdown attenuates TGF‐*β*‐induced (H) and CTGF‐induced (J) COL1A1 upregulation, *n* = 3, *p < 0.05.

HEYL, HEY1, and HES1 are effectors of the Notch pathway in fibrosis promotion^[^ ^17^, ^18^ ^]^ and are up‐regulated upon Notch signaling ligands binding to NOTCH receptors.^[^ ^19^, ^20^ ^]^ Here, we studied the expression of these genes during meniscus development. HEYL was only expressed in smooth muscle cells and pericytes during embryonic stages (E24 and E35 weeks) and hardly expressed at other stages or in other cell populations (**Figure** **7**A,B), suggesting that HEYL may play pivotal role in the critical stage of meniscus fibrosis. Continuous histological sections showed consistent expression of HEYL and NOTCH3 in mouse meniscus (Figure S7, Supporting Information). We also examined the effect of NOTCH3 knockdown on HEYL expression in vivo and found a significant reduction in HEYL expression upon NOTCH3 knockdown (Figure 7C,D).

**Figure 8:**
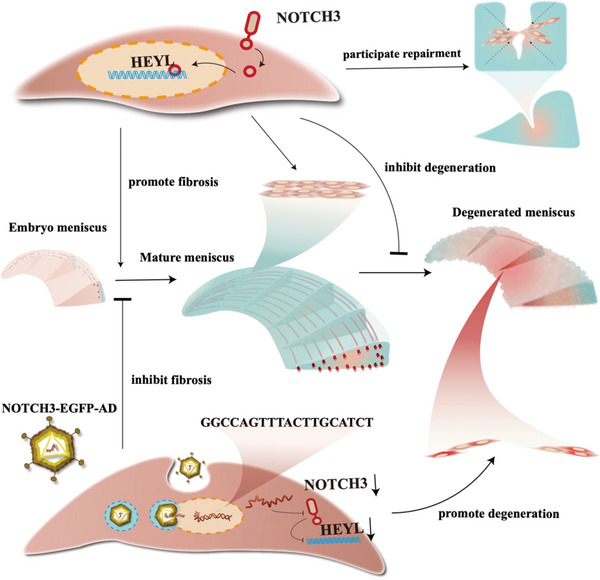
Diagrammatic sketch of physiological and functional characterization of smooth muscle cells in the meniscus.

TGF*β* ^[^ ^21^ ^]^ and CTGF^[^ ^22^ ^]^ promote fibrosis by NOTCH signaling pathway activation. Therefore, TGF*β*1 and CTGF were used to activate NOTCH signaling in vitro. First, human healthy meniscus cells were treated with TGF*β*1, NOTCH3 siRNA (or HEYL siRNA), or TGF*β*1+NOTCH3 siRNA (or HEYL siRNA). The COL1A1 RNA levels were detected by qRT‐PCR. TGF*β*1 upregulated COL1A1 expression and NOTCH3 siRNA per se downregulated COL1A1. TGF*β*1 effect was attenuated with the concomitant exposure to either NOTCH3 siRNA or HEYL siRNA (Figure 7E–G). We also stimulated human healthy meniscus cells using CTGF and obtained similar results (Figure 7I,J). Overexpression of NOTCH3 in vitro showed ascending expression of HEYL and COL1A1 (Figure S8, Supporting Information). Altogether, these results illustrate that HEYL, as the downstream target of NOTCH3, plays a key role in meniscus fibrosis process (**Figure** **8**).

## 3 Discussion

The existence of vessels in the meniscus was proverbial, but vessel‐related cells such as endothelial cells and smooth muscle cells were not incorporated into the commonly described menisci cell types. Therefore, studies relative to the functions of these cells beyond blood supply were non‐existent.^[^ ^23^ ^]^ Current research accepted that meniscus mainly contained fibroblast like cells and chondrocytes like cells, and although recent studies identified meniscus progenitor cells,^[^ ^6^, ^24^, ^25^ ^]^ the phenotypic characteristics of these cells as well as of their ancestors remained unclear. In our study, we found that human mature meniscus progenitors derive from embryonic smooth muscle cells that are not only distributed around blood vessels, but also on meniscus surface. This distribution was consistent with previous descriptions for meniscus progenitor cells.^[^ ^4^, ^24^ ^]^

Smooth muscle cells originate from multiple stem cells types.^[^ ^26^ ^]^ During embryogenesis, smooth muscle cells recruitment is a determinant step for the vascular system formation.^[^ ^27^ ^]^ In the adult, smooth muscle cells act as a progenitor cell pool to continually repair vessel injuries.^[^ ^28^ ^]^ Furthermore, smooth muscle cells have been shown to play an important role in tissue fibrosis. For instance, the aberrant proliferation of smooth muscle cells increased periarterial fibrosis and consequently promoted pulmonary hypertension.^[^ ^29^, ^30^ ^]^ Smooth muscle cells repression relieved pulmonary hypertension through fibrosis inhibition.^[^ ^31^ ^]^ In a meniscus injury model, smooth muscle cells proliferated and migrated to the injury site to promote repair.^[^ ^6^, ^32^ ^]^ In this work, we found that repressing smooth muscle cells inhibited fibrosis progression in immature meniscus but accelerated degeneration in the mature meniscus, suggesting that smooth muscle cells can be a target for fibrosis promotion and meniscus degeneration prevention, useful for tissue engineering.

Smooth muscle fibers mainly line the circulatory and digestive systems, and like other muscle fibers, their main function is to contract.^[^ ^33^ ^]^ However, smooth muscle does not contract or release as quickly as skeletal or cardiac muscle but provide consistency and elastic tension.^[^ ^34^ ^]^ In our study, smooth muscle fibers were mainly distributed at the base and surface of the healthy mature meniscus oriented circumferentially rather than radially. In degenerated meniscus, fibers were significantly reduced and presented a disorganized structure. Nonetheless, the fibers number increased at the site of meniscus injury repair. These results suggest that smooth muscle fibers play an important role in the maintenance of meniscus structure and function, as well as in injury repair. To our knowledge, this is the first study to systematically investigate the structure and function of meniscus smooth muscle fibers, which will provide a deeper understanding of meniscus dynamics.

Notch signaling is a highly conserved pathway involved in cell fate determination in embryonic development but also in vascular development, smooth muscle biology, and tissue fibrosis.^[^ ^35^ ^]^ Notch receptors, Notch1 and Notch4, are highly expressed in endothelial cells, while NOTCH3 is predominantly expressed in smooth muscle cells.^[^ ^19^ ^]^ Our single cell sequencing analysis of human meniscus cells showed NOTCH3 specific high expression in smooth muscle cells, with a constant expression pattern throughout the embryonic stage to adulthood, which suggests that NOTCH3 can be used as a target gene for smooth muscle cells. NOTCH3 signaling has already been shown to be crucial for smooth muscle cell proliferation and function.^[^ ^36^, ^37^ ^]^ In our study, we knocked down NOTCH3 in vivo and found a significant reduction in the number of both smooth muscle cells and fibers. Furthermore, these alterations preceded a delay in meniscus fibrosis, suggesting that NOTCH3 signaling in the meniscus smooth muscle cells regulates fibrosis progression. Due to NOTCH3 large sequence, overexpression in vivo is currently not available. However, we have managed the overexpression experiment in vitro which left us an anticipated result.

Notch activation is required for joint development and articular cartilage maintenance, however, its effect on osteoarthritis is highly complex. Hosaka et al. found that intra‐articular injection of Notch inhibitor prevented osteoarthritis progression,^[^ ^38^ ^]^ whereas Lin et al. found that Notch1 knockdown exacerbated osteoarthritis.^[^ ^39^ ^]^ In our study, 26 and 52 weeks‐old mice were injected with NOTCH3‐EGFP‐AD, and while the menisci showed markedly degeneration 8 weeks later, there was no obvious osteoarthritis progression. This might be related to the study model, since destabilization of the medial meniscus model can quickly lead to osteoarthritis, but non‐traumatic osteoarthritis is a slow progress. While NOTCH3 activation was shown to promote chondrogenic differentiation,^[^ ^40^ ^]^ suggesting that NOTCH3 activation in knee joint may protect cartilage and inhibit meniscus degeneration, its effect on osteoarthritis still requires further studies.

In summary, we identified the presence of smooth muscle cells in the meniscus, elucidating the distribution characteristics of the smooth muscle fibers and revealing smooth muscle cells as precursors of fibrochondrocyte progenitors. We also demonstrated that NOTCH3 inhibition in meniscus smooth muscle cells prevented meniscus fibrosis and exacerbated degeneration in a HEYL‐dependent manner. Altogether, our study highlighted the important mechanisms of meniscus fibrosis and degeneration, providing experimental evidence and targets for future therapeutic approaches.

## 4 Experimental Section

### Human Meniscus Samples

The acquisition of human meniscus was approved by the ethical committee of Sun‐Yat Sen Memorial Hospital (2020‐126). Written informed consent was obtained from the individual(s) and minor(s)’ legal guardians/next of kin for the publication of any potentially identifiable images or data included in this article. Human meniscus embryonic samples were obtained from nine patients who proceeded with abortion owing to fetal malformations. Tissue was collected after ensuring that there was no alteration to the musculoskeletal system in the embryos. Healthy menisci were harvested from four teenagers requiring amputation due to severe trauma (mean ± SEM age, 13.5 ± 4.6 years). Human osteoarthritis samples were obtained from six patients (mean ± SEM age, 60.5 ± 3.8 years) who were undergoing knee arthroplasty surgery.

### Histology Staining

Histological staining analyses were performed according to manufacturer's instructions. HE staining kit (G1003; Servicebio) was used for HE staining, Saffron Fast Green dye solution kit (G1053; Servicebio) for Safranin fast green staining, and Masson staining solution (G1006; Servicebio) for Masson staining.

Immunofluorescence (IF) and immunohistochemistry (IHC) analyses were conducted according with protocols.^[^ ^1^ ^]^ Primary antibodies for IHC included: NOTCH3 (bioss‐1812R, 1:200), *α*‐SMA (Proteintech, No.14395‐1‐AP, 1:3000), COL1A1 (Proteintech, No.67288‐Ig, 1:2500), COL2A1 (Proteintech, No.28459‐1‐AP, 1:800), PLVAP (bioss‐12737R, 1:50), and HEYL (Proteintech, 15679‐1‐AP, 1:200). Primary antibody for IF included fast myosin (GB112130, red light, 1:1000), slow myosin (GB112131, green light, 1:1000), NOTCH3 (bioss‐1812R, 1:200),HEYL (Proteintech, 15679‐1‐AP, 1:200), and MYOCD (bioss‐9472R, 1:200). Section area and positive area quantifications were conducted using Image J software.

### Single‐Cell RNA Statistical Analysis

This work applied fastp^[^ ^2^ ^]^ tool with default parameter filtering of the adaptor sequence and removed the low‐quality reads to obtain clean data. The UMI‐based^[^ ^3^ ^]^ clean data were mapped to the human genome (Ensemble version 91) using STAR41^[^ ^4^ ^]^ mapping with customized parameters from the UMI‐tools standard pipeline to obtain UMI counts of each sample. The Seurat package (version 2.3.4, https://satijalab.org/seurat/) was used for cell normalization and regression based on the expression table according to the UMI counts of each sample and the percentage of mitochondria rate to obtain scaled data.

Then, single‐cell trajectory analysis was applied using Monocle2 (http://cole‐trapnell‐lab.github.io/monocle‐release) and DDR‐Tree and default parameters. Based on the pseudo‐time analysis and branch expression analysis modeling (BEAM analysis), a branch fate‐determined gene analysis was performed.

### Animal Experiments

The animal study design was reviewed and approved by the Ethical Committee of Sun Yat‐sen Memorial Hospital (2020‐B0270).

C57BL/6 pregnant mice (E13) were used for this study. Briefly, each mouse was anesthetized with 1.5% isoflurane in an induction chamber at a respiratory rate of 60 breaths per min, then removed from the induction chamber and placed in a respiratory mask infused with 0.5%–1% isoflurane in oxygen. The mouse abdomen was shaved, and a 2 cm long incision was made along the abdominal skin and peritoneum around the position of the womb. The uterine wall was held open with tweezers and the embryos were extracted from the uterus, using tweezers to lift the uterine wall.

For knockdown experiments, NOTCH3‐EGFP‐AD were prepared with sequence (GGCCAGTTTACTTGCATCT) before which knockdown effect has been confirmed. The promoter for this virus was U6 and EGFP was used as a tag. The adenoviruses were stored at −80 °C with titer over 1× 1011 PFU per mL.

For the in vivo NOTCH3 knockdown experiment, 10 µL of NOTCH3‐EGFP‐AD adenovirus with titer over 1× 1011 PFU per mL combined with 2 µL polybrene (50 ug mL^−1^) were introduced in the amniotic cavity along with warm ampicillin in PBS (2 mL of 0.1 mg mL^−1^) to prevent infection. The peritoneum and abdominal skin were sutured from the inside to the outside, and a 1% lidocaine solution was applied to the wound for pain relief. One week after birth, 10 µL of the adenovirus at the same concentration (together with polybrene) were injected through the patellar ligament. Three weeks after birth, the injection was repeated and samples harvested for the P21d group. For each experimental timepoint, mice in the control group were operated with the same procedure but injected with a control adenovirus. Four weeks after birth, all mice were sacrificed and samples harvested.

For the study of NOTCH3 in fibrosis development, C57BL/6 mice at 26 and 52 weeks of age were used. At each timepoint (26 weeks or 52 weeks), mice were anesthetized using induction chamber and respiratory mask and injected with the NOTCH3‐EGFP‐AD through the patellar ligament, according to the previous procedure. Control group was operated with the same procedure but injected with a control adenovirus. Injection was repeated every 2 weeks after the first one. Eight weeks after the first injection, mice were sacrificed and samples harvested. The knee joint pain after meniscus injury was evaluated in mice after first injection using von Frey filaments. Mouse was placed on a wire‐mesh platform (Excellent Technology Co.) to restrict their move. During the test, a set of von Frey fibers (Stoelting Touch Test Sensory Evaluator Kit #2 to #9; ranging from 0.015 to 1.3 g force) were applied to the plantar surface of the hind paw and then held for 3 s when the fiber bowed. The threshold force required to elicit withdrawal of the paw (median 50% withdrawal) was evaluated five times on each hind paw with sequential measurements separated by at least 5 min.

### Isolation of Meniscus Cells and Cell Culture

Human mature meniscus was cut into small pieces and digested with 4 mg mL^−1^ protease for 1 h, followed by 0.25 mg mL^−1^ collagenase P for 4 to 6 h. After washing with 0.04% bovine serum albumin (BSA) in phosphate‐buffered saline (PBS), the cell pellets were re‐suspended in fresh 0.04% BSA and filtered through a 35 µm cell strainer. Dissociated single cells were then stained for viability assessment using Calcein‐AM (Thermo Fisher Scientific) and Draq7 (BD Biosciences). Primary human meniscus cells were cultured in DMEM/F ‐12 medium (0 2753; Gibco), according to the supplier's protocol. All experiments were performed using cells within three passages.

### Meniscus Cells Treatment

Knockdown of NOTCH3/HEYL in human meniscus cells was performed using siRNA. Meniscus cells were plated at 80% confluency per well in a 6‐well plate. siRNA duplexes using Lipofectamine Transfection Reagent (Gibco Life Technologies, USA) were prepared in OptiMEM (31 985 070; Thermo Fisher) according to the manufacturer's recommendations and used at a final concentration of 50 nm. A negative control siRNA was used for the control group. The cells were exposed to the duplex for 48 h. Knockdown efficiency was assessed through RT–qPCR for NOTCH3/HEYL expression. After transfection, meniscus cells were treated with the growth factors, TGF‐*β*/CTGF, diluted in PBS at 40 ng mL^−1^ for 48 h. Control group was treated with PBS. Cells were collected for RNA extraction and qRT‐PCR.

Overexpression of NOTCH3 in human meniscus cells was performed with plasmids carrying NOTCH3 mRNA searched from NCBI (NM_00435.3, [cds:6966 bp], [Protein:2321aa]). Transfection was following the procedure of LipoTrans Liposomal Transfection Regant (MhBio). 4ug of plasmid was blended with 250 µL DMEM, and another 250 µL DMEM was mixed with 6 µL LipoTrans. 5 min after stewing, 2 solutions were mixed and incubated for 20 min. Total 500 µL mixture of LipoTrans‐DNA was added into one well of 6‐plate and removed 6 h later with 2 mL new medium added in each well. Control group was going through the same procedure except for empty plasmid transfected. 48 h after transfection, cells were collected for RNA extraction and qRT‐PCR.

### RNA Extraction and qRT‐PCR

The extracted RNA was reverse transcribed into cDNA using Super‐Script III Reverse Transcriptase (Invitrogen). Quantitative real time PCR (qRT‐PCR) was performed using 2× SYBR Green Master Mix (Arraystar, Rockville, MD, USA) on an Applied Biosystems ViiA 7 Real‐time PCR System (Foster City, CA, USA). The final reaction system consisted of 1 µL of cDNA, 3.2 µL of double distilled water, 0.4 µL of forward and reverse primers, and 5 µL of 2× SYBR Green PCR Master Mix. The gene expression levels were quantified using the 2−ΔΔCt method. Each primer was designed based on sequences available at NCBI database:

**Table:** 

NOTCH3	F	TGGCGACCTCACTTACGACT
	R	CACTGGCAGTTATAGGTGTTGAC
HEYL	F	GGAAGAAACGCAGAGGGATCA
	R	CAAGCGTCGCAATTCAGAAAG
COL1A1	F	GAGGGCCAAGACGAAGACATC
	R	CAGATCACGTCATCGCACAAC
GAPDH	F	GGAGCGAGATCCCTCCAAAAT
	R	GGCTGTTGTCATACTTCTCATGG
MCAM	F	AGCTCCGCGTCTACAAAGC
	R	CTACACAGGTAGCGACCTCC

### Statistical Analyses

Statistical analyses were performed using GraphPad Prism software (GraphPad Prism Software). Data are presented as the mean ± standard deviation (SD) of the results of at least three independent experiments. Student's *t*‐test and Mann–Whitney U test were used to identify significant differences between groups. One‐way analysis of variance (ANOVA) and Kruskal–Wallis tests were used for comparisons between multiple groups. Statistical significance was set at *p* < 0.05.

## Conflict of Interest

The authors declare no conflict of interest.

## Supporting information

Supporting Information

## Data Availability

The data that support the findings of this study are available from the corresponding author upon reasonable request.
