# Supplementary material for: Silencing of NOTCH3 Signaling in Meniscus Smooth Muscle Cells Inhibits Fibrosis and Exacerbates Degeneration in a HEYL‐Dependent Manner
Source: Adv Sci (Weinh). 2023 Apr 7;10(16):2207020. doi: 10.1002/advs.202207020 (PMC10238196; doi:10.1002/advs.202207020)
Supplement: Supplementary file 1 — Supporting Information [file ADVS-10-2207020-s001.pdf]

ADVANCED SCIENCE
Open Access

Supporting Information

for *Adv. Sci.*, DOI 10.1002/advs.202207020

Silencing of NOTCH3 Signaling in Meniscus Smooth Muscle Cells Inhibits Fibrosis and Exacerbates Degeneration in a HEYL-Dependent Manner

Hao Sun, Fangzhou Liu, Zhencan Lin, Zongrui Jiang, Xingzhao Wen, Jie Xu, Zhiqi Zhang* and Ruofan Ma**

SUPPLEMENTARY MATERIALS

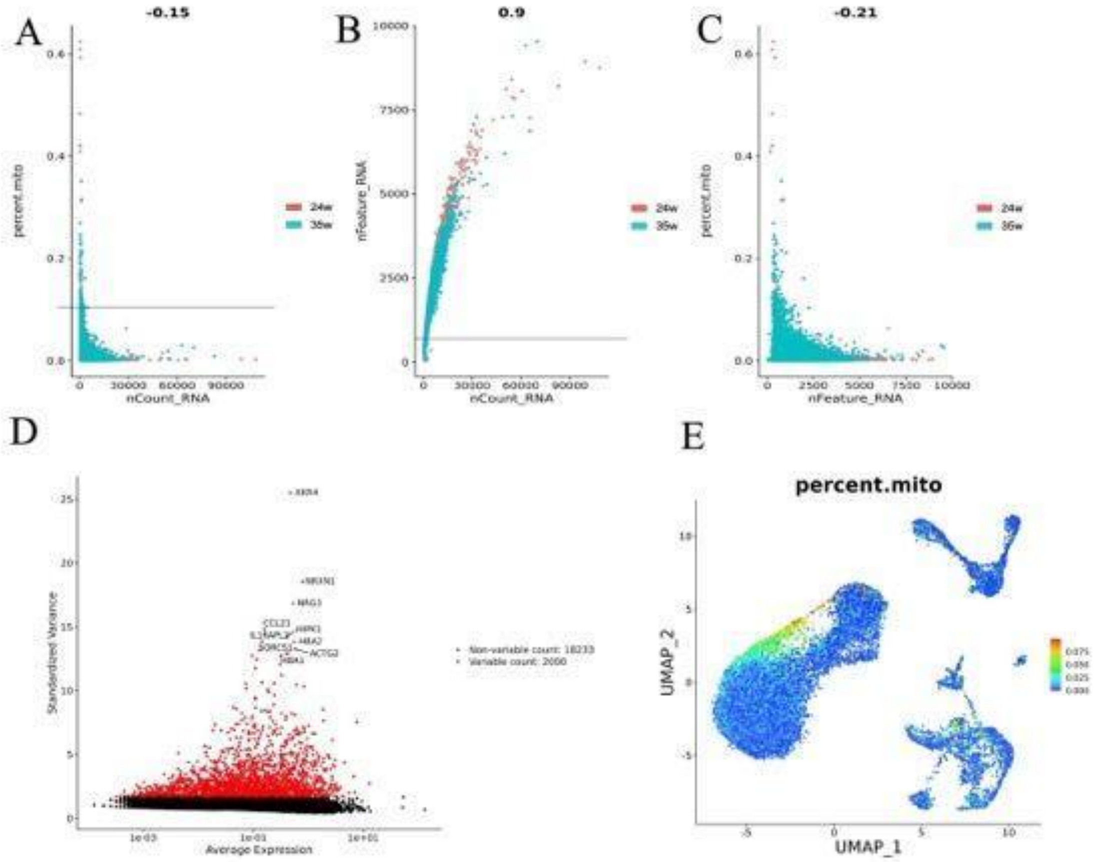

Figure S1 sc-RNA sequencing cell quality control (A) Mitochondria UMI (unique molecular identifier) rates in cell distribution. Cells with Mitochondria-UMI rate higher than 40% failed to pass the cell quality control. (B) Cells containing less than 200 expressed genes failed to pass the cell quality control. (C) Combination of Mitochondria UMI rates and Cell gene expression. (D) 2000 Variable gene was counted in further analysis and 18233 gene remain ignorance for lower value. (E) Mitochondria distribution in Uniform manifold approximation and projection (UMAP) results indicate cell quality.

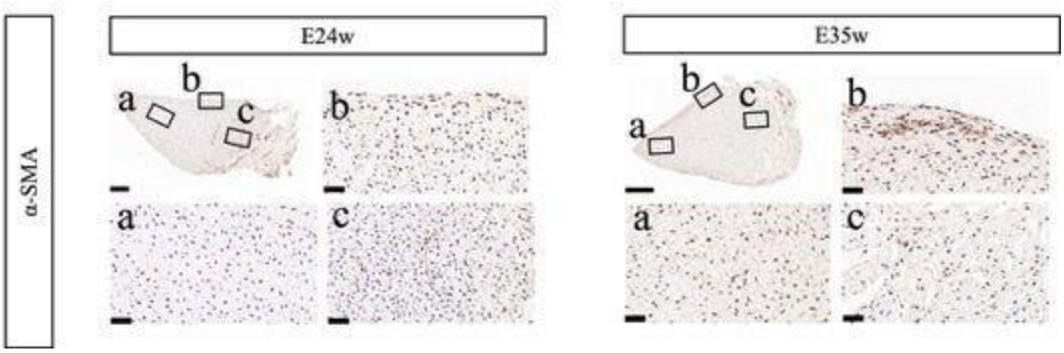

Figure S2 α-SMA distribution in embryo meniscus. Immunohistochemistry (IHC) of α-SMA at E24w and E35w meniscus. SMA levels indicate smooth muscle cells presence. Scale bar: 200 μm for 24 and 35 weeks; 20 μm for magnified picture. n=3.

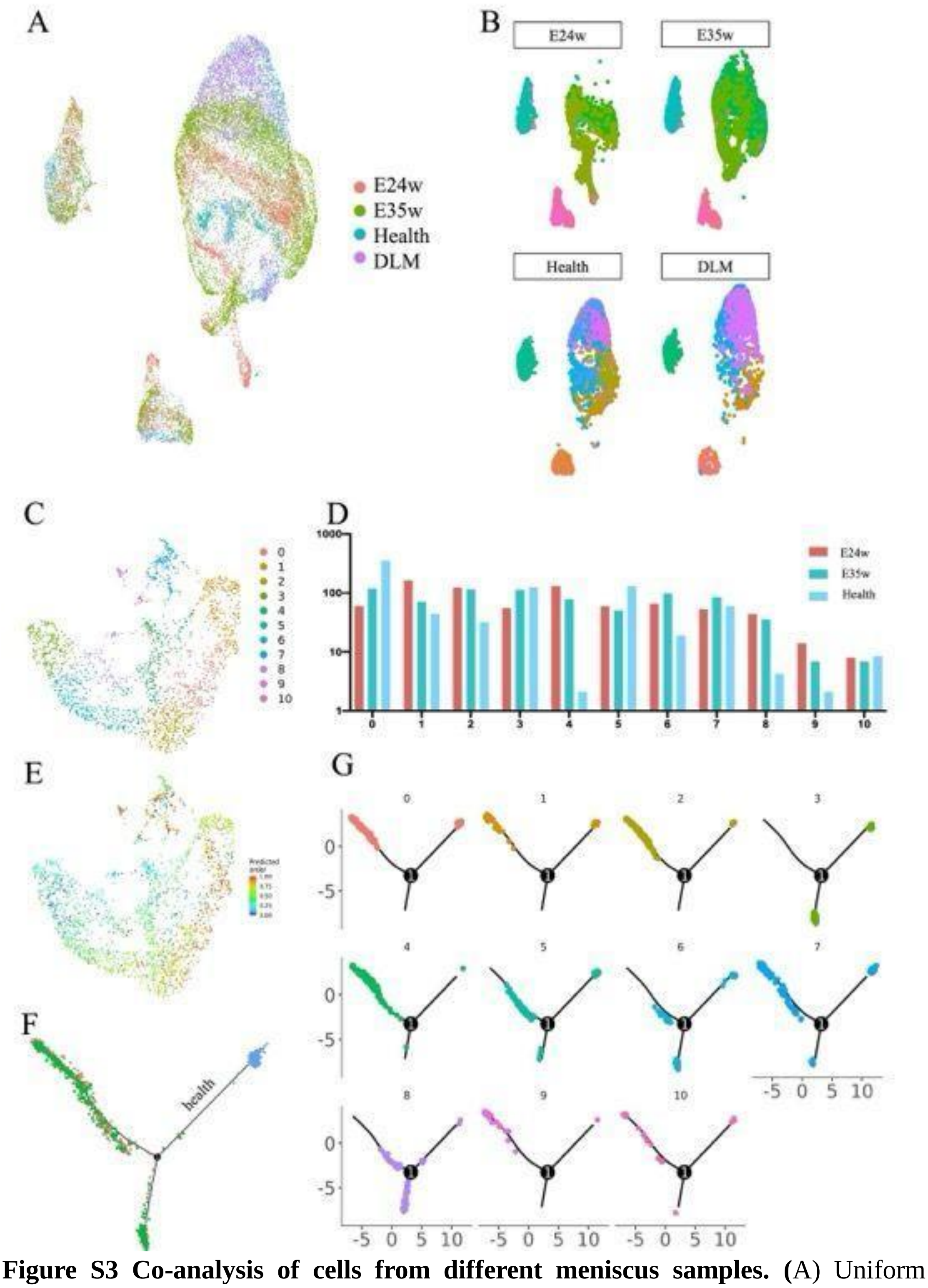

Figure S3 Co-analysis of cells from different meniscus samples. (A) Uniform manifold approximation and projection (UMAP) results of different meniscus samples including: E24w, E35w, healthy meniscus, and discoid lateral meniscus. Three major groups are highlighted namely fibroblast and chondrocyte, endothelial cell, and smooth muscle cell and fibrochondrocyte progenitors (FCP). (B) UAMP distribution of different cell groups in each sample type. (C) UMAP results of co-analysis of FCP in adult and smooth muscle cell/pericyte (SMC/PC) in embryo meniscus, present well-distributed FCP in SMC/PC. (D) Cell number count of different clusters in each

sample type. (E) UMAP results of cells distribution according to differentiation capacity determined by Cyto-trace. (F, G) Pseudo-time analysis of FCP and SMC/PC (F), and distribution of different clusters in the Pseudo-time analysis (G).

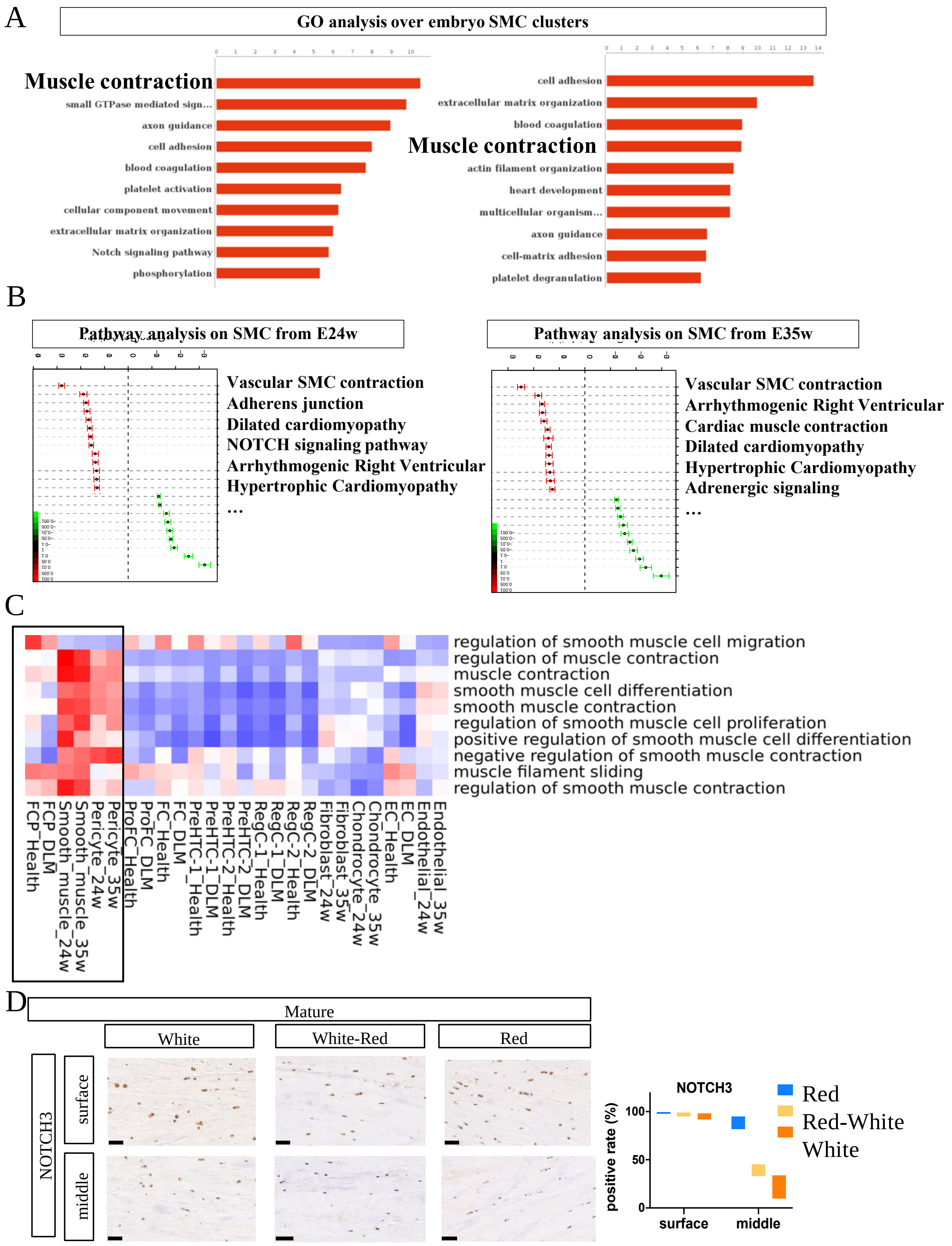

Figure S4 Pathway analysis of SMC and FCP and NOTCH3 distribution in coronal section. (A) GO analysis of SMC clusters in embryo samples revealed function of muscle contraction. (B) Pathway analysis on SMC from E24w and E35w. (C) muscle related pathway analysis over combined all 4 samples. (D) NOTCH3 expression in a coronal section of a mature meniscus. Scale bar: 50 μm, n=6.

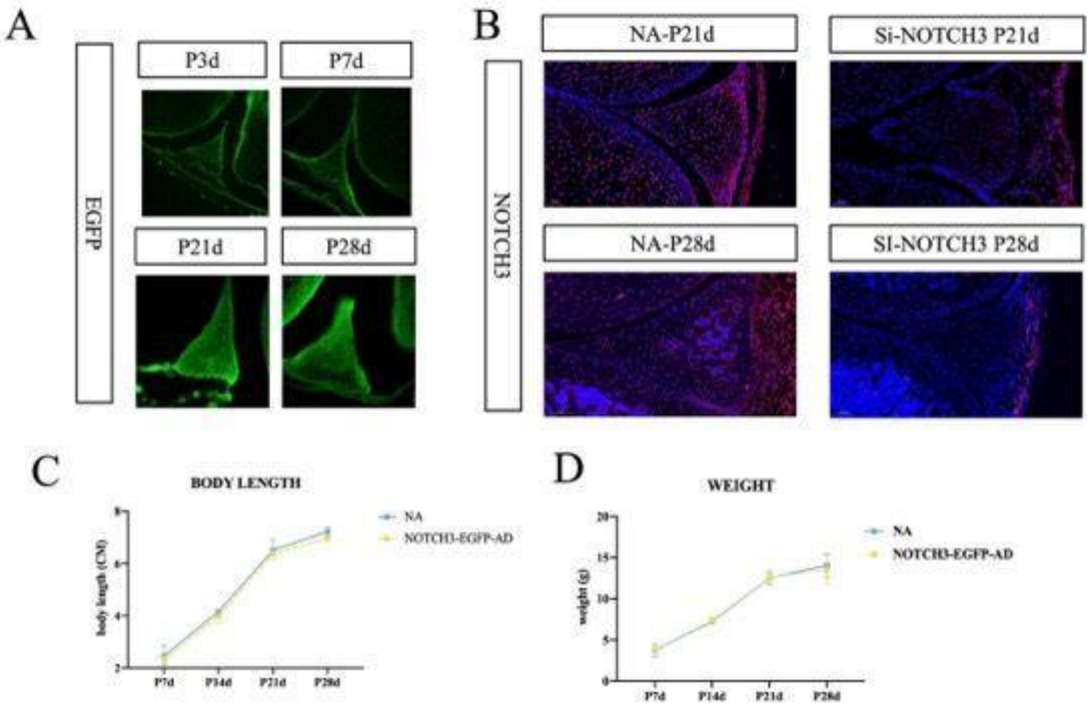

Figure S5 NOTCH3-knockdown in embryo meniscus. (A) Positive expression of EGFP in meniscus confirmed infection. (B) Immunofluorescence of NOTCH3 in the mouse meniscus after NOTCH3-knockdown. Scale bar: 100 μm. n=3. (C, D) No significant changes in body length (C) and weight (D) observed between NA (control group) and NOTCH3-knockdown, n=6.

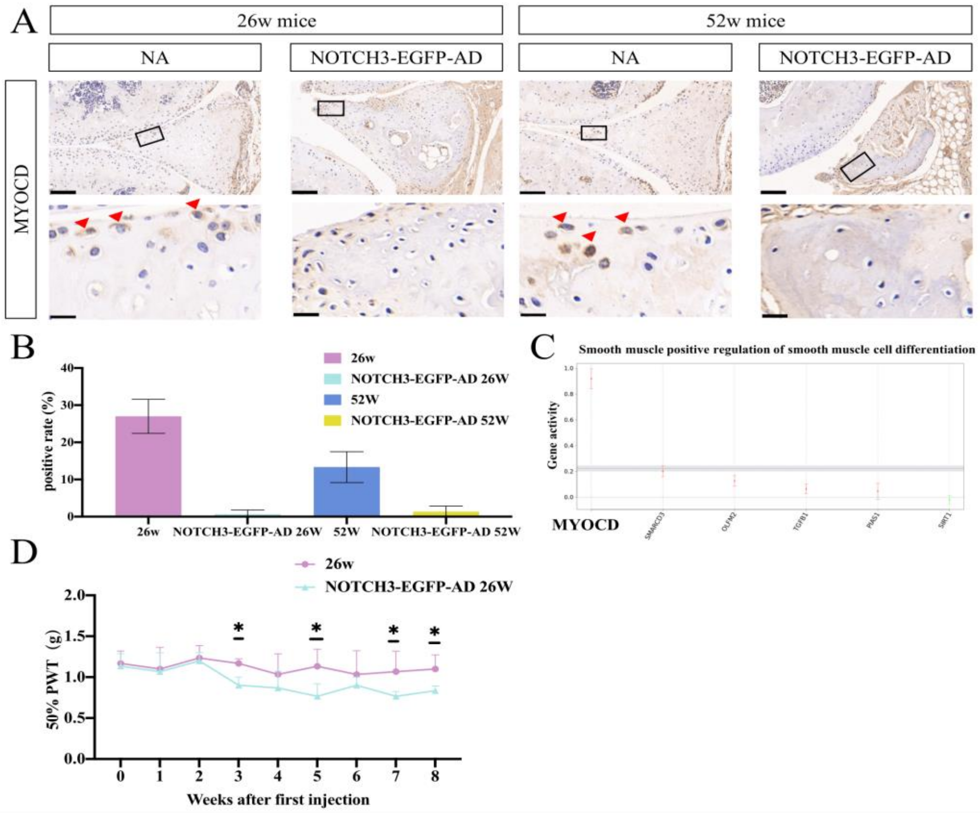

Figure S6 NOTCH3-knockdown in aged mice (A) IHC of MYOCD in aged and NOTCH3 knockdown mice. Scale bar: 100 μm for overview; 20 μm for magnified pictures, n=3. (B) Positive rate of MYOCD in aged mice and NOTCH3 knockdown mice. n=3, *p<0.05. (C) Gene activity analysis of smooth muscle regulation of smooth muscle cell differentiation revealed importance of MYOCD. (D) Pain withdrawal threshold of aged mice after NOTCH3-EGFP-AD injection tested. n=3/groups. *p<0.05.

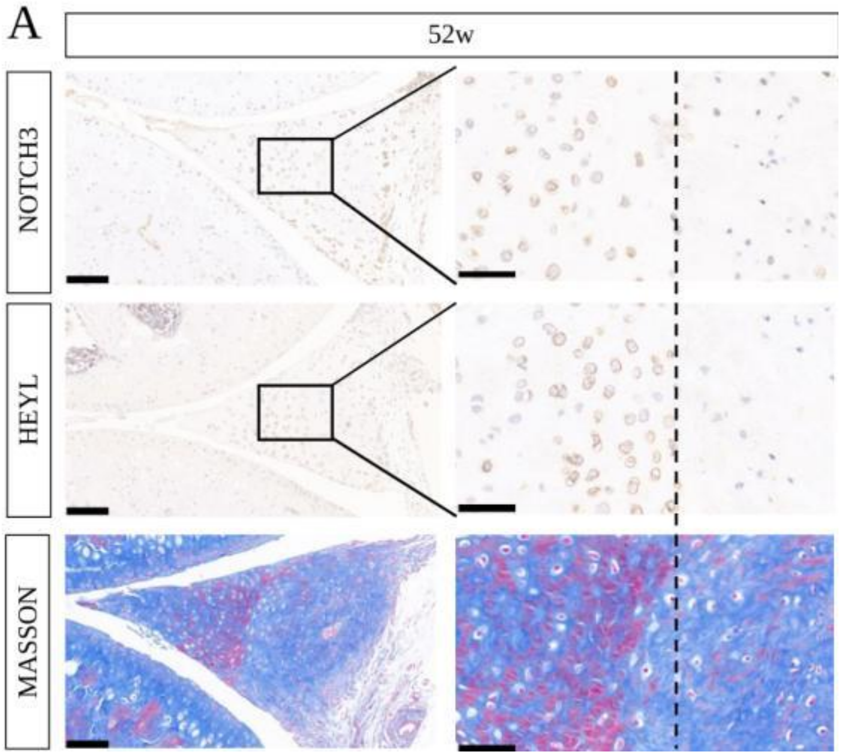

Figure S7 Co-labelling of NOTCH3 and HEYL in the meniscus. Masson staining and immunohistochemistry (IHC) results of NOTCH3 and HEYL indicate co-expression these two proteins in the mouse meniscus. Scale bar: 100 μm for overview; 50 μm for magnified pictures, n=3.

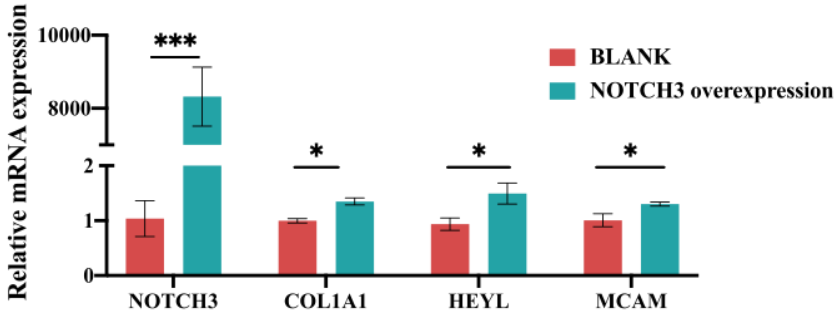

Figure S8 Overexpression of NOTCH3 in degenerated meniscus cells. mRNA expression level of NOTCH3, COL1A1, HEYL after NOTCH3 overexpression, n=3, *p<0.05.
